# Annotations

**Published:** 1887-10-29

**Authors:** 


### Annotations.

Lady Doctors in France.

French politeness has recently been put to the test, and found wanting. Mademoiselle Blanche Edwards, a fully qualified doctor of medicine, having become a candidate for a hospital surgeonship, an attempt has been made by several members of the Faculty to have her disqualified, on the ground of her having passed by a few months the limit of age fixed for the competitors for such appointments. If it were the custom for the Faculty to adhere strictly to the letter of the law in bestowing such appointments, protest would be useless ; but it is a common thing in the case of male candidates to overlook the fact of their being more than the statutory twenty-eight years of age, and only last year another female candidate, Mademoiselle Augusta Klumpke, who was more than twenty-eight years old, was appointed surgeon to the Toureine Hospital. The position taken up by the opponents of Mademoiselle Edwards is, therefore, both illogical and unjust. Their manner of expressing their disapproval of her candidature, by creating an uproar and crying out—"A la porte blanche!" "Vive la limite d'age !" is a kind of bearish rudeness of which only French politeness can be guilty. Mingled with these exclamations were shouts of "Vive Boulanger !" "A bas Ferron !" and other political watchwords, totally irrelevant to the question on hand. The reason of this apparently meaningless demonstration is that Mademoiselle Edwards' cause is supported by M. Spuller, the Minister of Education, and her male colleagues have made their opposition to her an opportunity for expressing their dislike to the reigning ministry. But beyond this lies a more personal and practical cause for anger. The French male doctors are jealous of medical women, who are proving themselves no mere frivolous triflers with science, but conscientious students who mean to be, if possible, successful practitioners. It is, in fact, a matter of trade competition. There is a demand for lady doctors, but, if none are to be had, male physicians will be accepted as substitutes. The male practitioners are therefore tempted to take any pretext that presents itself to put their female rivals out of the field. But if the Faculté de Médecine do not desire women to practise in France, they should not permit them to study and graduate at the University of Paris. A medical diploma is not a mere ornamental degree ; it is a practical instrument which every man who receives it means to use in earning his living. Women regard it in the same light ; and to put this tool into their hands, and then forbid them to use it, is the height of unreason as well as of injustice.

The Dangers of the Dentist's Chair.

The well-stuffed velvet-covered chair which the dentist provides for the victims of toothache is meant for a solace, a dumb assurance to the sufferer that he will not have to endure one unnecessary pang, or even an avoidable discomfort. Whether the unwilling visitors who occupy the seat are grateful for this considerate attention is somewhat doubtful, and it is certain that they would do their best to avoid it if they suspected that the luxurious head-rest might inflict on them a disease more insidious than toothache, and equally objectionable. Not long ago, the eldest son of a family, where the children are carefully kept, and are not allowed to mingle with playfellows less well looked after than themselves, developed an attack of ringworm. The source of the disease could not be traced, and while it was still a subject of conjecture, the second boy had, for some reason, to consult a dentist. A few days afterwards the ringworm appeared on him, and it was then remembered that a visit to the same operator had preceded the development of the disease on his elder brother. The ringworm had been contracted in both cases by the contact of the child's head with the back of the dentist's chair. Doctors who devote their attention to skin diseases know that such accidents are by no means uncommon ; but it is almost certain that dentists themselves are unconscious that their furniture is the means of conveying infection. The danger could easily be avoided by covering the back of the chair with a small antimacassar, giving each patient a fresh one. The same result could be attained by using a piece of American cloth, which can easily be washed, as a protection for or from the chair ; but as a matter of appearance a white linen antimacassar is decidedly preferable ; and most dentists will be glad to use such a simple precaution, rather than incur a danger which must ultimately injure their practice. No one will return where they have once caught such a disease as ringworm. In this connection it may be mentioned that equal care for their customers' safety is required from hair-dressers, a case of ringworm on the neck having been known to result from the contact of one of those voluminous shroud-like aprons in which they drape their customers. These things should never be used for more than one person without being well washed.

Truth and Justice—and Houses to let.

A simple-minded gentleman recently became tenant of a cottage in the West Highlands on the faith of an advertisement which described it as being situated two hundred yards from the sea, and commanding a splendid view of Ben Cruachan and Lady Margaret's Tower. The interior of the cottage offered sufficient comfort, and the tenant was promised the use of a boat. Arrived at this attractive-sounding residence, he found that of three bedrooms only two were habitable, the other being a closet too small to contain a bed. The promised boat was absent altogether ; but this mattered less, as the cottage was situated, not two hundred yards, but a mile from the sea. There were, however, some trenches filled with water not more than a few yards from the house ; but these suggested rowing less distinctly than malaria. The "splendid view" included neither Ben Cruachan, the sea, nor Lady Margaret's Tower ; it consisted solely and entirely of bog, diversified with stacks of peat. Naturally, the gentleman who had taken the cottage declined to occupy it, alleging that the advertisement which induced him to do so was false and misleading. The landlord and author of the said advertisement regarded the matter otherwise, and sued the tenant for a month's rent. The case was tried in the Edinburgh Sheriff's Court, and judgment was given for the landlord, the Sheriff remarking, as a reason for his verdict, that "when people took houses in the West Highlands they must expect the descriptions to be somewhat highly coloured." The incident clearly brings out that the landlord of this cottage, and the author of a description which casts such a glamour over an incommodious and insanitary dwelling, is lost to the world in a lonely corner of the West Highlands. In view of his indubitable imaginative powers, and the revival of the public taste for romance, he ought to devote himself to legitimate fiction, and cast "the light that never was on sea or land" over Patagonia or the Antarctic Regions, or any other place which people like to read of and don't want to live in. We are not prepared to suggest another sphere of usefulness (?) for the Sheriff ; but it is evident that he, too, is not at present in the right place. Our readers who live south of the Tweed, and who take houses in Scotland in the summer, will not need the moral in their case to be here pointed.

